# Unlocking Optimal Strategies: A Systematic Review Exploring the Efficacy of Physical Exercise vs Cognitive Training for Enhancing Executive Functions in Mild Cognitive Impairment and Dementia

**DOI:** 10.1192/bjo.2024.158

**Published:** 2024-08-01

**Authors:** Geetanjali Grover, S.M. Edney, Saadia Tayyaba

**Affiliations:** Cardiff University, Wales, United Kingdom

## Abstract

**Aims:**

While there is research on physical exercise and cognitive training on cognitive improvement in older adults, there is none comparing these two interventions for their efficacy on executive functioning specifically in the population with a diagnosis of Mild Cognitive Impairment (MCI) or dementia. This study aims to bridge this gap and determine the superiority between the two interventions to enhance executive functions among individuals with MCI or dementia. Besides establishing evidence for the benefits of these socially prescribed interventions, it also aims to highlight their differential effects on executive functions. Additionally, it seeks to evaluate the feasibility of implementing these interventions to provide evidence-based insights that inform clinical practice.

**Methods:**

Sixteen randomised control trials were meticulously selected using the Cochrane selection manual and PRISMA guidelines from an extensive search across prominent academic databases. Stringent quality assessment was conducted for each study using the modified Centre for Reviews and Dissemination checklist, Jadad and PEDro scales and the Cochrane Risk of Bias tool ensuring methodological rigour. The studies provided a total of 1593 participants with a mean age of 74.36 (SD = 5.54), randomly allocated in various intervention groups. Each study was critically appraised, analysed and the findings presented as a narrative synthesis and a meta-analysis performed with the available data.

**Results:**

Physical exercise showed statistically insignificant improvement on the Stroop Test (p = 0.19) while no significant correlation was seen in Verbal Fluency (p = 0.032). Cognitive Training intervention had a significant improvement in both Stroop test (P = 0.0009) and Verbal Fluency (p = 0.00). The study also found that diverse contextual and personal factors like socioeconomic levels, education, personal preferences, general health conditions, mood, dependence on others, and genetics, are some factors that influence an individual's response to intervention and hence determine its efficacy.

**Conclusion:**

There is limited statistical evidence to conclude the superiority of one intervention over the other. However, this systematic review highlights that the effectiveness of an intervention cannot be assessed solely on its statistical effect size. Rather, one must go beyond numerical assessments for a comprehensive understanding of individual circumstances that may pose barriers to engagement with the interventions, thus influencing their acceptability and effectiveness. A holistic and multidimensional perspective of the disease with a personalised intervention plan may be the new solution.

